# A Case of Coombs-Negative Hemolytic Anemia Prompting Diagnosis of SARS‐CoV‐2

**DOI:** 10.7759/cureus.20034

**Published:** 2021-11-30

**Authors:** Thanuja Neerukonda, Walid Moughabel, Justin Chen, Devansh Patel, Faiza Manji

**Affiliations:** 1 Internal Medicine, Brandon Regional Hospital, Brandon, USA; 2 Hematology and Oncology, Brandon Regional Hospital, Brandon, USA

**Keywords:** sars-cov-2, - hematology, coombs' negative, coronavirus disease 2019 (covid-19), hemolytic anaemia

## Abstract

Case reports have discussed coronavirus disease of 2019 (COVID-19) patients presenting with hemolytic anemia, specifically with a positive direct antiglobulin test. However, Coombs-negative hemolytic anemia in COVID-19 patients has been rarely reported. We present an unusual case of Coombs-negative hemolytic anemia caused by the severe acute respiratory syndrome coronavirus 2 (SARS-CoV-2) which responded with evidence-based COVID-19 treatments. We demonstrate the importance of considering SARS-CoV-2 as a cause of Coombs-negative hemolytic anemia, and we illustrate how treatment of the underlying COVID-19 illness, even if it is just supportive care, will help resolve the associated hemolysis.

## Introduction

Coronavirus disease 2019 (COVID-19) caused by severe acute respiratory syndrome coronavirus 2 (SARS‐CoV‐2) was declared a pandemic in March, 2020 by the World Health Organization [1]. Symptomatic COVID-19 cases have ranged from mild to critically ill [2]. It has been shown that patients with a more severe disease have a higher chance of developing hematologic abnormalities, specifically coagulopathies [3]. Hemolytic anemia has been previously shown to be associated with neoplasms, medications, genetic causes, infections, and oxidant drugs [4]. Case reports have discussed COVID-19 patients presenting with hemolytic anemia, specifically with a positive direct antiglobulin test for either IgG, C3d, or both IgG and C3d [5-10]. However, Coombs-negative hemolytic anemia in COVID-19 patients has been only reported twice in literature [11,12]. We present an unusual case of Coombs-negative hemolytic anemia caused by SARS-CoV-2 which responded with evidence-based COVID-19 treatments established by the National Institutes of Health (NIH) COVID-19 Treatment Guidelines Panel in the United States of America.

## Case presentation

A 23-year-old African American male with history of heroin and 3,4-methylenedioxymethamphetamine (MDMA) use presented with symptoms of nausea, vomiting, and hematuria. At the time of admission, he was afebrile, blood pressure 126/57 millimeters of mercury (mmHg), heart rate 79 beats/minute, and oxygen saturation 96% on room air. Physical exam showed dry mucosal membranes; regular heart rate and rhythm; breath sounds clear to auscultation; abdominal tenderness in the right upper quadrant. Initial labs included white blood cell count of 8.3 x 103 µl, hemoglobin of 10.8 g/dL, hematocrit 32.1%, mean corpuscular volume (MCV) 94.4 fL, red cell distribution width (RDW) 13.5%, total bilirubin of 3.4 mg/dl, direct bilirubin 0.5 mg/dl, creatinine of 1.79 mg/dL, creatinine kinase 450 U/L, normal aminotransferases, ferritin 3510 ng/mL, and D-dimer 1897 ng/mL. Urinalysis was significant for 100 mg/dL protein, large blood, and 11-20 RBCs. Computed tomography (CT) abdomen/pelvis and abdominal ultrasound were normal. The visualized lung bases seen on CT abdomen/pelvis were unremarkable. The following day, our patient’s hemoglobin dropped to 7.6 g/dL. Additional labs were ordered including reticulocyte count: 5.32%, lactate dehydrogenase (LDH): 1704 U/L, and haptoglobin: <20 mg/dL.

Our patient’s elevated reticulocyte count, elevated LDH, elevated indirect bilirubin, decreased haptoglobin, and acute drop of hemoglobin were concerning for hemolytic anemia. Our differential diagnosis was very broad upon time of admission and included- autoimmune hemolytic anemia, drug-induced hemolytic anemia, congenital/hereditary disorders, thrombotic microangiopathy (TMA), and viral or bacterial infectious etiologies. Direct antiglobulin test (DAT) polyspecific, detecting IgG and complement and indirect coombs testing were negative. Patient’s urine drug screen was negative for amphetamines, opiates, cocaine, and barbiturates. Blood alcohol level was < 3 mg/dL. Glucose-6-phosphate dehydrogenase deficiency, hereditary spherocytosis, and sickle cell disease were considered but peripheral blood smear showed normal red blood cell morphology without bite cells, spherocytes, or schistocytes. Blood smear without schistocytes and no noted thrombocytopenia made TMA unlikely; a disintegrin and metalloproteinase with thrombospondin type 1 motifs, member 13 (ADAMTS13) level was 55%, not consistent with thrombotic thrombocytopenic purpura (TTP). Hepatitis profile and human immunodeficiency virus (HIV) testing were negative. Initially, COVID-19 was not suspected as the patient did not experience shortness of breath, cough, fevers, or chills. However, as we were in the midst of the COVID-19 pandemic and viral infections are known to be a cause of Coombs-negative hemolytic anemia, on day 2 of hospitalization SARS‐CoV‐2 was tested and resulted positive.

Supportive treatments for COVID-19 were started immediately including albuterol sulfate inhaler, tiotropium bromide inhaler, incentive spirometry, as needed ant-emetics, and folic acid daily. Based on the NIH Treatment Guidelines Panel, our patient's COVID-19 illness was categorized as mild given no hypoxia or requirement of supplemental oxygen [13]. These guidelines recommend against the use of all corticosteroids in patients with mild disease as there is currently a lack of safety and efficacy data and systemic glucocorticoids may actually cause harm in these patients. Remdesivir has insufficient evidence for or against patients who do not require supplemental oxygen, and baricitinib is not recommended in these patients either [13]. Thus, dexamethasone, IL-6 inhibitors, JAK inhibitors, and remdesivir were not given to our patient.

As treatments were continued, additional diagnostic workup was also completed. Plasma cell dyscrasia was considered in the differential; immunoglobulins, urine protein electrophoresis, and serum protein electrophoresis were all normal. Both Vitamin B12 and folate levels were normal, ruling out any megaloblastic anemia. Anti-nuclear antibody pattern was noted to be positive and anti-centromere antibody was mildly elevated at 1.2, nonspecific for any autoimmune disease. All other autoimmune testing was normal including rheumatoid factor, cryoglobulin, cytoplasmic-ANCA antibody, anti-myeloperoxidase, JO-1 antibody, SS-A/Ro antibody, SS-B/La antibody, smith antibody, SM/RNP IgG antibody, double-stranded DNA antibody, complement C3 and C4, glomerular basement antibody, chromatin antibody, and scl-70 scleroderma antibody. Initially, drug-induced hemolytic anemia was considered high on our differential. However, urine drug screen was negative and the patient denied any use of medications or exposure to toxins. MDMA has been associated with TMA, but we reiterate that TMA was not suspected given the lack of significant thrombocytopenia and no schistocytes were seen on peripheral blood smear. Hematologic malignancies seemed unlikely in our 23-year-old patient as he complained of no B-cell lymphoma symptoms and peripheral blood smear was normal. Myelodysplastic syndrome (MDS) was also unlikely given the patient’s age and absence of pancytopenia. Thus, bone marrow biopsy was not obtained.

With supportive COVID-19 treatments, the patient’s hemolytic labs started to improve as seen in Table 1. Upon time of discharge, the patient was hemodynamically stable and his symptoms completely resolved. With the guidance of consultant services, hematology and nephrology, the patient was diagnosed with Coombs-negative hemolytic anemia secondary to SARS‐CoV‐2.

**Table 1:** Course of hemolysis labs during hospitalization.

Laboratory Test	Time of Admission	Day 1	Day 2	Day 3	Day 4	Normal Range
Hemoglobin (g/dL)	10.8	7.6	7.5	8.2	8.9	13.7-17.5
Hematocrit (%)	32.1	24.5	23.1	25.3	26.5	40.1-51.0
MCV (fl)	94.4	103.4	99.6	97.3	95.3	79.0-92.2
Reticulocyte Count (%)	-----	4.78	5.32	5.87	7.03	0.51-1.81
Absolute Reticulocyte Count (10^6^/uL)	-----	0.129	0.124	0.154	0.196	0.026-0.095
Immature Reticulocyte Fraction (%)	-----	18.40	21.70	26.10	27.50	0.00-16.1
Platelets (10^3^/uL)	197	190	178	234	283	15-450
Lactate Dehydrogenase (U/L)	-----	1704	1192	966	849	87-241
Haptoglobin (mg/dL)	-----	< 20.0	-----	-----	-----	30-200
Total Bilirubin (mg/dL)	3.4	3.5	1.1	0.6	0.3	0.2-1.0
Direct Bilirubin (mg/dL)	0.5	-----	0.80	-----	-----	0.0-0.2
Aspartate Aminotransferase (U/L)	168	130	46	25	18	15-37
Alanine Aminotransferase (U/L)	22	18	19	19	19	16-61
Alkaline Phosphatase	85	71	70	58	94	45-117

## Discussion

Coombs-negative hemolytic anemia is characterized by laboratory evidence of hemolysis plus a negative Coombs test. Various viral infections have been associated with Coombs-negative hemolytic anemia including hepatitis A virus, hepatitis E virus, cytomegalovirus, and influenza [14]. A negative Coombs-test result in patients with hemolytic anemia after a viral infection could be explained by a few mechanisms including low-affinity antibodies undetected by the commonly used assays, low antibodies on red cell membranes, or autoantibodies like IgA or IgM [15]. The SARS-CoV-2 spike protein-CD147 interaction may also explain how the virus can invade red blood cells as it also plays a role for the invasion of erythrocytes by P. falciparum [16]. Thus, intravascular hemolysis may be a complication of COVID-19. Typically, when we see hemolytic anemia, our differential diagnosis should include viral infections. Other viral infections besides Hepatitis and HIV were not tested but our patient showed laboratory evidence of hemolytic anemia with a negative Coombs test, initiating our work up for SARS‐CoV‐2. One of the mainstays of treatment for Coombs-negative hemolytic anemia is steroid therapy [14]. NIH Treatment Guideline Panel recommended against all steroid therapy in patients with mild COVID-19 disease. Nonetheless, patients receiving corticosteroids for an underlying condition are recommended to continue their therapy. Steroid treatment in our patient was initially held, and we decided to monitor the patient overnight and if hemolysis continues to persist, steroid therapy would then be initiated. Subsequently, his hemolytic anemia and clinical symptoms improved with supportive COVID-19 treatments. Therefore, steroid therapy was not given and we believe that our patient had Coombs-negative hemolytic anemia secondary to SARS‐CoV‐2.

To our knowledge, only two studies on Coombs-negative hemolytic anemia associated with SARS‐CoV‐2 have been published in the literature. Guido et al. tested 38 SARS-CoV-2 positive patients and evaluated plasma hemoglobin (plasma-hb) levels in the evaluation of hemolysis [11]. They found 15 out of 38 patients had levels at 15 mg/dL but only nine out of the 38 patients had highly pathogenic levels, greater than 30 mg/dL. Only one patient was DAT positive and six out of those nine patients did die compared with the patients with levels below 30 mg/dL. This illustrates how hemolysis may be an under-recognized complication of SARS-CoV-2. However, 61% of these patients were mechanically ventilated, experiencing a severe version of COVID-19, so it is difficult to say if deaths were from COVID-19 itself or a complication of hemolysis. COVID-19 treatments including hydroxychloroquine, azithromycin, convalescent plasma, tocilizumab, gimsilumab, and remdesivir were variously used based on the patient’s clinical status. In comparison, Bae et al.’s patient was initially treated with hydroxychloroquine, azithromycin, and ceftriaxone for COVID-19 [12]. On hospital day 2, he received a three-day course of IVIG which subsequently improved the hemolysis. It is unclear if the patient’s rapid improvement was due to IVIG or a reflection of spontaneous recovery. Our patient had a milder course of COVID-19 in comparison to both these cases and supportive COVID-19 treatments were all that were needed to improve the hemolysis. We do believe that treating the underlying illness will help resolve the hemolytic anemia. However, the severity of the COVID-19 illness and treatment-resistant hemolytic anemia may require additional treatments such as glucocorticoids, IVIG, or rituximab. Therefore, additional research efforts need to be made to investigate treatment options for Coombs-negative hemolytic anemia secondary to SARS‐CoV‐2.

The aim of this case report was to acknowledge that even though hemolytic anemia associated with SARS-CoV-2 have been reported previously, it may also present as Coombs-negative hemolytic anemia. SARS-CoV-2 should be considered in the differential for the cause of hemolytic anemia, Coombs-negative or positive. We demonstrate how the acute management of COVID-19 in milder cases will resolve Coombs-negative hemolytic anemia. However, further research efforts are needed to identify moderate-severe COVID-19 cases and evaluation of additional treatment options if COVID-19 treatments, including steroids, do not improve hemolysis.

## Conclusions

This case presentation demonstrates the importance of considering SARS-CoV-2 as a cause of Coombs-negative hemolytic anemia. We illustrate how treatment of the underlying illness, COVID-19, will resolve the associated hemolytic anemia prior to considering glucocorticoids, IVIG, or rituximab. If hemolysis does persist despite COVID-19 treatment, further research efforts need to be increased to evaluate the success rates of other hemolytic anemia treatment modalities in these patients.

## References

[1] (2021). WHO Timeline ‐ COVID‐19. World Health Organization. https://www.who.int/news-room/detail/29-06-2020-who-covidtimeline.

[2] Zhu N, Zhang D, Wang W (2020). A novel Coronavirus from patients with pneumonia in China, 2019. N Engl J Med.

[3] Tang N, Li D, Wang X, Sun Z (2020). Abnormal coagulation parameters are associated with poor prognosis in patients with novel coronavirus pneumonia. J Thromb Haemost.

[4] Narla J, Mohandas N (2017). Red cell membrane disorders. Int J Lab Hematol.

[5] Lazarian G, Quinquenel A, Bellal M (2020). Autoimmune haemolytic anaemia associated with COVID-19 infection. Br J Haematol.

[6] Lopez C, Kim J, Pandey A, Huang T, DeLoughery TG (2020). Simultaneous onset of COVID-19 and autoimmune haemolytic anaemia. Br J Haematol.

[7] Capes A, Bailly S, Hantson P, Gerard L, Laterre PF (2020). COVID-19 infection associated with autoimmune hemolytic anemia. Ann Hematol.

[8] Jacobs J, Eichbaum Q (2021). COVID-19 associated with severe autoimmune hemolytic anemia. Transfusion.

[9] Hindilerden F, Yonal-Hindilerden I, Akar E, Yesilbag Z, Kart-Yasar K (2020). Severe autoimmune hemolytic anemia in COVID-19 infection, safely treated with steroids. Mediterr J Hematol Infect Dis.

[10] Maslov DV, Simenson V, Jain S, Badari A (2020). COVID-19 and cold agglutinin hemolytic anemia. TH Open.

[11] Lancman G, Marcellino BK, Thibaud S, Troy K (2021). Coombs-negative hemolytic anemia and elevated plasma hemoglobin levels in COVID-19. Ann Hematol.

[12] Bae JY, Jeon JE, Hussein KI, Lee MS (2021). Coombs-negative hemolytic anemia in a male with COVID-19. Clin Case Rep.

[13] (2021). COVID-19 Treatment Guidelines Panel. Coronavirus Disease 2019 (COVID-19) Treatment Guidelines. National Institutes of Health. https://www.covid19treatmentguidelines.nih.gov.

[14] Gilliland BC (1976). Coombs--negative immune hemolytic anemia. Semin Hematol.

[15] Kamesaki T, Kajii E (2018). A comprehensive diagnostic algorithm for direct antiglobulin test‐negative autoimmune hemolytic anemia reveals the relative ratio of three mechanisms in a single laboratory. Acta Haematol.

[16] Crosnier C, Bustamante LY, Bartholdson SJ (2011). Basigin is a receptor essential for erythrocyte invasion by Plasmodium falciparum. Nature.

